# A model of chronic, transmissible Otitis Media in mice

**DOI:** 10.1371/journal.ppat.1007696

**Published:** 2019-04-10

**Authors:** Kalyan K. Dewan, Dawn L. Taylor-Mulneix, Laura L. Campos, Amanda L. Skarlupka, Shannon M. Wagner, Valerie E. Ryman, Monica C. Gestal, Longhua Ma, Uriel Blas-Machado, Brian T. Faddis, Eric T. Harvill

**Affiliations:** 1 Department of Infectious Diseases, College of Veterinary Medicine, University of Georgia, Athens, Georgia, United States of America; 2 Center for Vaccines and Immunology, College of Veterinary Medicine, University of Georgia, Athens, Georgia, United States of America; 3 University of Colorado Hospital, Aurora, Colorado, United States of America; 4 Department of Microbiology, Franklin College of Arts and Sciences, University of Georgia, Athens, Georgia, United States of America; 5 Department of Pathology, Athens Veterinary Diagnostic Laboratory, College of Veterinary Medicine, University of Georgia, Athens, Georgia, United States of America; 6 Department of Otolaryngology-Head and Neck Surgery, Washington University School of Medicine, St. Louis, Missouri, United States of America; University of Toronto, CANADA

## Abstract

Infection and inflammation of the middle ears that characterizes acute and chronic otitis media (OM), is a major reason for doctor visits and antibiotic prescription, particularly among children. Nasopharyngeal pathogens that are commonly associated with OM in humans do not naturally colonize the middle ears of rodents, and experimental models in most cases involve directly injecting large numbers of human pathogens into the middle ear bullae of rodents, where they induce a short-lived acute inflammation but fail to persist. Here we report that *Bordetella pseudohinzii*, a respiratory pathogen of mice, naturally, efficiently and rapidly ascends the eustachian tubes to colonize the middle ears, causing acute and chronic histopathological changes with progressive decrease in hearing acuity that closely mimics otitis media in humans. Laboratory mice experimentally inoculated intranasally with very low numbers of bacteria consistently have their middle ears colonized and subsequently transmit the bacterium to cage mates. Taking advantage of the specifically engineered and well characterized immune deficiencies available in mice we conducted experiments to uncover different roles of T and B cells in controlling bacterial numbers in the middle ear during chronic OM. The iconic mouse model provides significant advantages for elucidating aspects of host-pathogen interactions in otitis media that are currently not possible using other animal models. This natural model of otitis media permits the study of transmission between hosts, efficient early colonization of the respiratory tract, ascension of the eustachian tube, as well as colonization, pathogenesis and persistence in the middle ear. It also allows the combination of the powerful tools of mouse molecular immunology and bacterial genetics to determine the mechanistic basis for these important processes.

## Author summary

Bacterial infections of the middle ears (otitis media), particularly among children, is a global problem, causing fever, pain, conductive hearing loss and possible complications due to persistence/recurrence. Current understanding is poor, and advances are slowed by limitations of experimental systems that, for example, inject large numbers of human pathogens into the middle ears of rodents. Such approaches introduce tissue damage, bypass progression from natural colonization to disease and these pathogens fail to persist as chronic infections, the period when complications associated with otitis media begin to increase. Here we report that *Bordetella pseudohinzii*, a gram-negative bacterium we previously identified as a respiratory pathogen of mice, naturally and extremely efficiently colonizes and persists in their middle ears, causing acute and chronic histopathological changes that closely mimic otitis media in humans. Laboratory mice experimentally infected with low bacterial numbers by droplet inhalation consistently have their middle ears colonized and subsequently transmit the bacterium to cage mates. The natural *B*. *pseudohinzii-*mouse model provides an experimental system in which to study aspects of the progression from earliest colonization, infection, spread, pathogenesis to long-term persistence as well as providing the tools of immune-deficient mice that can reveal the contributions of each aspect of immunity in controlling and clearing chronic middle ear infection.

## Introduction

Acute otitis media (AOM) an inflammation of the middle ear caused by bacterial infections [1] affects a large portion of the human population every year and is a source of considerable morbidity and societal burden [2, 3]. The majority of those afflicted are infants and children, and the condition is a major reason for antibiotic prescription [4, 5]. The pathogenesis of OM is predominantly attributed to middle ear infections by transmissible pathogens that initially colonize the oro/nasopharynx [6, 7, 8]. Poorly understood mechanisms allow pathogens to ascend the Eustachian tube to reach and colonize the middle ear. Early stages of AOM often induce fever and pain caused by the accumulation of an inflammatory exudate in the middle ear [1]. Increased pressure on the tympanic membrane can affect the auditory ossicles housed in the middle ear resulting in a decrease in the acuity of conductive hearing [9]. AOM is usually a self-resolving condition however, in a subset of patients persistent infections lead to chronic otitis media (COM), a condition accompanied with the risk of more serious complications.[10] Limitations of tractable methods to study OM has severely limited our progress in understanding of the mechanisms used by pathogens to reach the middle ear, or of the host factors [11–14] that determine susceptibility to acute or chronic OM [10]. Therefore, while there has been progress in interventions that alleviate the suffering [15], the details of host-pathogen interactions during the establishment of acute OM and its progression to a chronic form remain elusive.

The study of OM has been primarily advanced by experiments in rodents, including chinchillas, gerbils, ferrets and rats [16, 17], in which lesions resembling those of acute human OM are recognizable. Experiments in such outbred laboratory animals are useful for evaluation of vaccines and interventions but are of limited use for detailed genetic and immunological characterization of host responses. In contrast, the mouse model has the benefit of widely available reagents (e.g. antibodies to every molecule) and well-established inbred populations with defined mutant and conditional mutant strains defective in a library of nearly all known immunological mechanisms, allowing their contributions to pathogenesis, immune response and protection to be evaluated [18–21]. However, human bacterial pathogens do not naturally colonize rodents and when delivered intranasally do not consistently reach the middle ear. To overcome this, large numbers of pathogens are directly inoculated into the middle ear by trans-bullar/tympanic injections [17]. This invasive procedure can cause tissue injury and inflammation in itself and can also have unintended consequences such as the untargeted spread of the pathogen. Furthermore, as this procedure entirely by-passes colonization of the nasopharynx and ascendance of the pathogen up the Eustachian tube to colonize the middle ear, it does not permit the study of these crucial stages. Finally, human pathogens inoculated into rodents using these invasive methods do not persist as chronic infections. Current methods to induce and study Chronic OM in animal models include rather severe treatments such as ligation of the Eustachian tubes by surgical manipulation [22, 23] or use of mutant mice to investigate genetic correlates of the disease [24, 25, 26].

In view of these limitations, there is a clearly defined need for a natural mouse pathogen that induces AOM/COM to investigate, i.) how pathogens ascend the eustachian tube, ii) the induction and progression of the pathology in the middle ears for the acute and chronic stages of otitis media, iii) the complexities of host immunity that determine disease outcomes and iv) treatments and interventions for the condition. A pathogen isolated from the respiratory tracts of laboratory mice and identified as *Bordetella hinzii* [27], was subsequently discovered to be a novel species and named *Bordetella pseudohinzii* [28, 29]. This mouse pathogen [30] has now been found to be circulating within multiple laboratory mouse colonies and wild rodents [31–34].

Here we show that intra nasal inoculation of mice with *B*. *pseudohinzii*, or its natural transmission from cage-mates, led to its rapid ascension of the Eustachian tube, substantial inflammation of the middle ear and progressive hearing loss. Colonization persisted for months, establishing a chronic infection in the middle ear accompanied by inflammation as well as bone resorption and remodeling. Furthermore, we noted that *B*. *pseudohinzii* has a very low infectious dose expected of a natural pathogen and naturally transmitted within cages from mouse to mouse, efficiently colonizing the respiratory and auditory systems. T and B cell-deficient mice had exacerbated infection, revealing roles for components of the adaptive immune system and demonstrating the potential of this mouse-as-natural-host experimental system to define the contributions of many specific immune functions in the control and clearance of bacterial infections of the middle ear. The *B*. *pseudohinzii* mouse host-pathogen system we describe here therefore allows the detailed study of both bacterial and immunological aspects of how a respiratory pathogen initially colonizes the nasal cavity, ascends to the middle ear, progresses to establish chronic middle ear pathology and transmits to new hosts.

## Results

### *Bordetella pseudohinzii* colonizes the middle ears of mice

**Fig 1:**
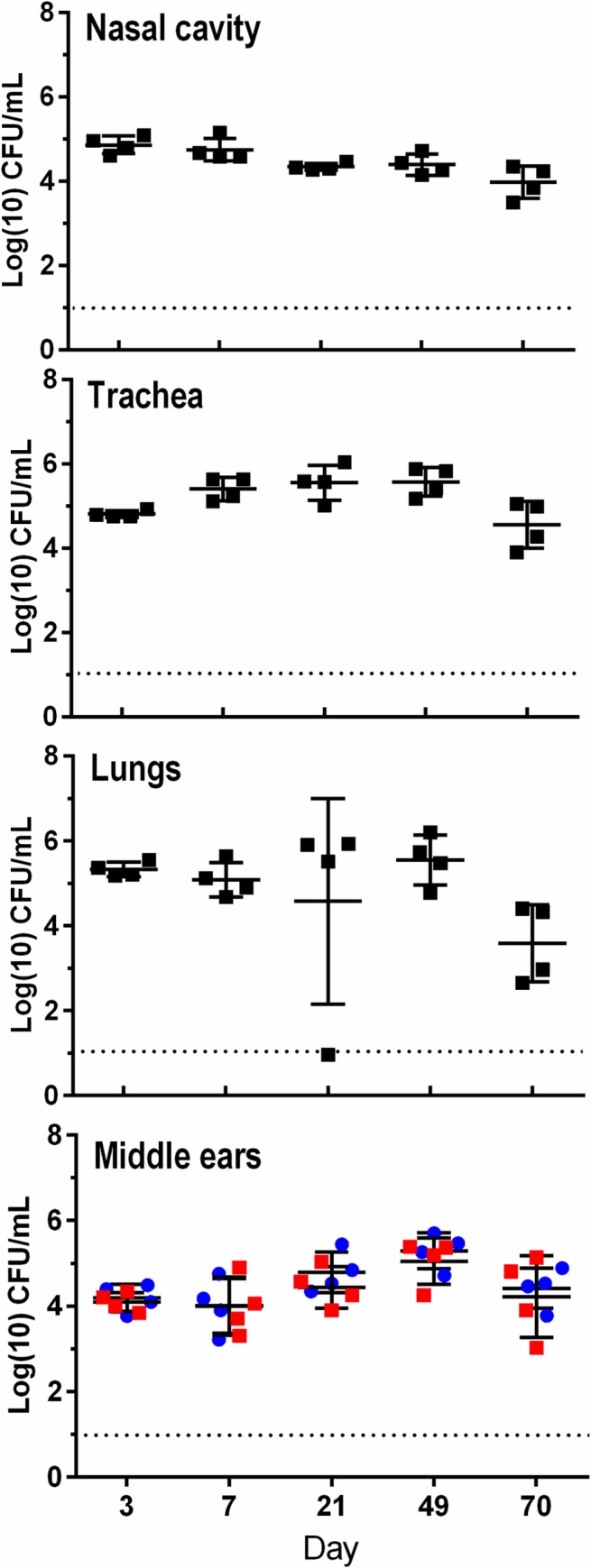
B. pseudohinzii colonizes the respiratory tract and middle ears of C57BL/6J mice. 1. Mice inoculated with 7,500 CFU of B. pseudohinzii (n = 4 per group) were sacrificed at days 3, 7, 21, 49, and 70 post inoculation. B. pseudohinzii numbers recovered from the nasal cavity, trachea, lungs and left and right middle ear bullae are presented for each day. Bacteria in the middle ears were bilaterally distributed in equal numbers (lower panel, red squares- left ear; blue squares- right ear). Error bars indicate standard deviation. Dotted lines indicate limit of detection.

In the course of studying noise-induced hearing loss in mice we observed variations in the Auditory Brainstem Response (ABR) thresholds that indicated a decrease in hearing sensitivity of several untreated mice among our colonies. Investigations of the cause led to the association between increased ABR thresholds and middle ear colonization by *Bordetella pseudohinzii*. *B*. *pseudohinzii* was recently identified by our group as a natural pathogen of mice that robustly colonizes their respiratory tracts [27, 28]. To determine if this bacterium was colonizing the middle ears via the respiratory tract, we inoculated groups of C57BL/6J mice from their external nares with 7,500 CFU bacteria in 25 μl of phosphate buffered saline. At days 3, 7, 21, 49 and 70 we euthanized 4 mice each and assessed bacterial colonization in the nasal cavity, trachea, lungs and the left and right middle ear bullae (Fig 1). By 3 days post inoculation the numbers of bacteria recovered from the respiratory tract (nasal cavity, trachea and lungs) had reached about ten-times the inoculation dose, indicating that *B*. *pseudohinzii* had grown and spread efficiently through the respiratory tract of its host. Interestingly, the middle ears of all mice were also colonized by the bacterium as early as day 3 post inoculation. Both left and right bullae were uniformly colonized at levels comparable to the nasal cavity (>10^4^ CFU) and it persisted till at least day 70 in comparable numbers. These results show that *B*. *pseudohinzii* efficiently spreads from nasal inocula to colonize and persist in the middle ears of C57BL/6J mice. Following a similar inoculation regimen *B*. *pseudohinzii* was also observed to colonize the middle ears of BALB/cJ mice and C3/HeJ mice, indicating that the ability to establish an infection in the auditory system via the nasopharyngeal route was common to diverse mouse strains (S1 Fig).

### Pathogenicity of *B*. *pseudohinzii*

To assess the pathology accompanying *B*. *pseudohinzii* colonization and persistence in the mouse ears and surrounding organs, a histological evaluation of the nose, brain, and ear was conducted, examining the incidence, severity, and distribution of inflammation. Analysis was conducted on days 3, 14, 28 and 123. Table S1 (Supporting Information) summarizes the incidence and severity of significant findings which were limited to the nose and middle ear with no evidence of brain inflammation detected at any time.

**Fig 2:**
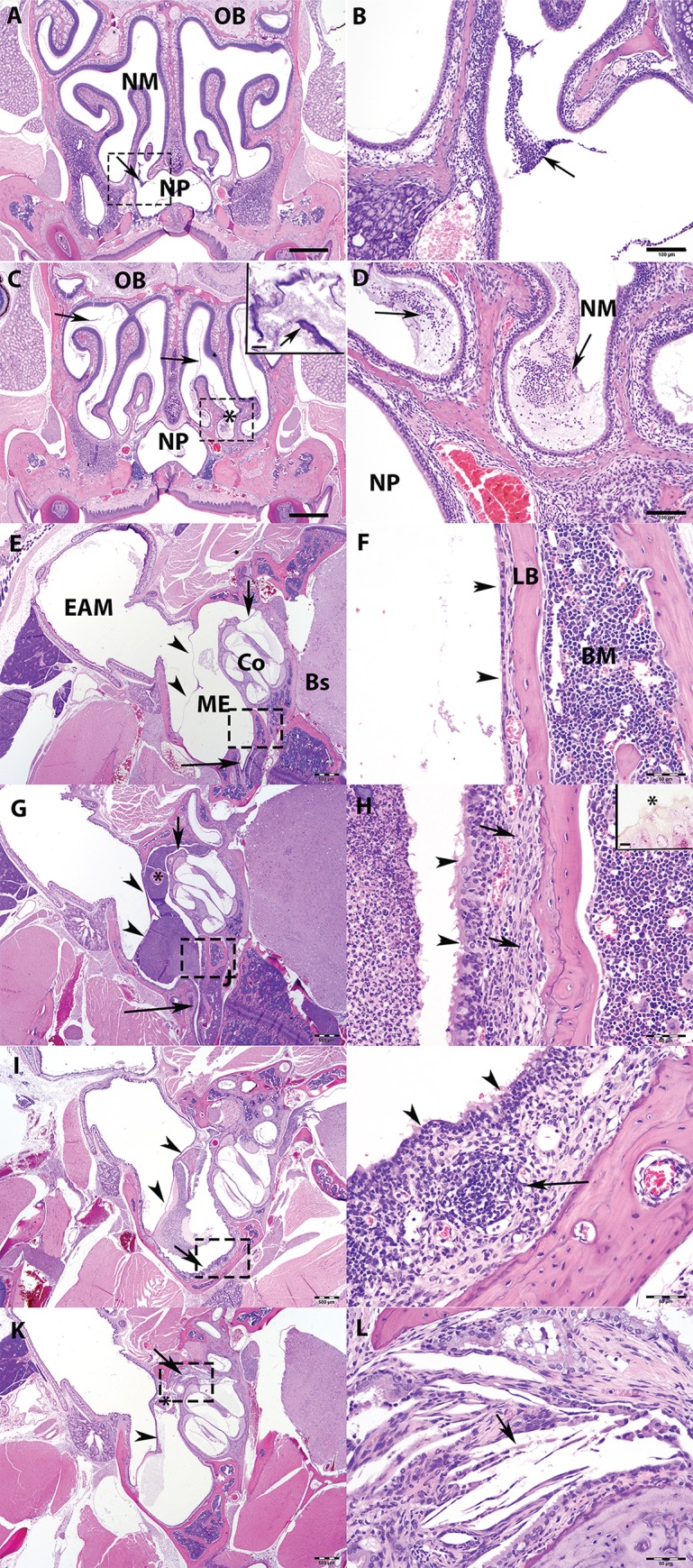
Histopathology of mouse nasal cavity and middle ears on Bordetella pseudohinzii colonization. Histopathology analyses conducted on C57BL/6J mice inoculated with 7,500 CFU of B. pseudohinzii delivered in 25 μl of PBS. Groups of infected mice (n = 6) were sacrificed on 3, 14, 28 and 123- days post inoculation (DPI). (A-D) Coronal sections of the nasal cavity of C57BL/6J mice stained with Hematoxylin and Eosin (HE) stain. 3 DPI. Image of nasal meatus showing small amounts of exudate at the frontonasal sinus (arrow). Scale bar = 500 μm. B) Higher magnification of Fig A (dashed rectangle) showing neutrophils embedded in pale basophilic mucopurulent exudate (arrow). Scale bar = 100 μm. C) 7 DPI. Image of nasal meatus with mild amounts of exudate (asterisk) and a thin basophilic, film-like layer extending throughout the nasal meatus (arrows and inset). Scale bar = 500 μm; Inset Scale bar = 10 μm. D) Higher magnification of Fig C, (dashed rectangle), showing mild amounts of neutrophils embedded in pale basophilic material (arrow). Scale bar = 100 μm. Abbreviations: Nasal meatus (NM); Olfactory bulb (OB); Nasopharynx (NP). (E-J) HE stained transverse sections through the middle ear of C57BL/6J mice controls (E, F) and infected with Bordetella pseudohinzii (G-J). E) 3 DPI showing Eustachian tube (long arrow), round window membrane (short arrow), and tympanic membrane (arrowheads). Scale bar = 500 μm. F) Higher magnification of E, (dashed rectangle area), showing the middle ear tympanic bulla and thin layer of ciliated epithelia (mucoperiosteum), overlying a thin lamina propria (arrowheads). Scale bar = 50 μm. G) 14 DPI showing large accumulation of exudate extending from Eustachian tube (long arrow), tympanic membrane (arrowheads), middle ear bones (asterisk), and round window (short arrow). Scale bar = 500 μm. H) Higher magnification of G, (dashed rectangle), Middle ear and tympanic bulla showing diffusely hypercellular mucoperiosteum (arrowheads) and hypertrophic mesenchymal cells (arrow) covering the lamellar bone (LM). Scale bar = 50 μm. Inset: Gram-negative (red) bacteria (asterisk) attached to cilia of the mucoperiosteum. Gram stain. Scale bar = 10 μm. I) 28 DPI showing moderate amounts of exudate in the middle ear and adjacent structures. Arrowhead points to the tympanic membrane, and arrow points to the mucoperiosteum. Scale bar = 500 μm. J) Higher magnification of Fig. I, (dashed rectangle), showing middle ear and tympanic bulla showing hypercellular mucoperiosteum (arrowhead) and lymphoid tissue formation (arrow). Scale bar = 50 μm. Abbreviations: External auditory meatus (EAM); Middle Ear (ME); Cochlea (Co); Brain Stem (BS); Lamellar bone (LB); Bone marrow (BM). (K-P) HE stained transverse sections through the middle ear of C57BL/6J mice 123 DPI with Bordetella pseudohinzii. K) Image shows focal granulomatous inflammation about the round window (arrowhead) and malleolus (asterisk). Tympanic membrane (arrow). Scale bar = 500 μm. L) Higher magnification of K, (dashed rectangle area), showing the round window with an area of granulomatous inflammation that contains cholesterol clefts (arrow) mixed with macrophages and neutrophils. Scale bar = 50 μm.

Coronal sections through the nasal cavity, at the level of the olfactory bulb indicated minimal to mild, multifocal suppurative rhinitis which persisted to day 28 (Fig 2A–2D) and varied from acute (predominantly neutrophils) to chronic (predominantly mixture of neutrophils, lymphocytes and plasma cells).

The first evidence of suppurative otitis media was visible by 3 days post inoculation (DPI). Infected mice exhibited minimal to severe, unilateral or bilateral suppurative otitis media, which varied from acute (3–14 DPI) to chronic (>28 DPI) (Fig 2E–2J). By day 14, an accumulation of exudate was seen extending from the Eustachian tube and filling the middle ear, pushing against the intact tympanic membrane, obscuring the middle ear bones, and extending through and displacing the round window membrane between the middle and inner ear (Fig 2G–2H). The mucoperiosteum became diffusely hypercellular, with increased numbers of neutrophils that extended into adjacent lamina propria. A layer of hypertrophic mesenchymal cells, likely osteoclasts and osteoblasts, covered the irregular, scalloped lamellar bone of the tympanic bulla. At day 28 (Fig 2I–2J) a moderate amount of exudate still remained in middle ear and adjacent structures. The mucoperiosteum remained diffusely hypercellular however, with fewer neutrophils but increased numbers of lymphocytes, with lymphoid tissue formations beginning to obscure the mucoperiosteum and adjacent lamina propria. No significant changes were noted on the walls of the adjacent cochlear bone or within the inner ear which remained intact throughout the length of the experiment. On day 123, granulomatous inflammation with formation of cholesterol clefts were observed, primarily about the round window and adjacent malleus bone (Fig 2K–2L). The tympanic membrane remained intact and Eustachian tubes unobstructed, while changes described earlier in the bony bulla had subsided, suggesting that the bone remodeling recognized earlier may be a reversible change. Altogether, histopathology findings indicate that *B*. *pseudohinzii* colonizes the mouse middle ear via the respiratory route (nasal cavity and Eustachian tube), establishing an acute inflammatory and chronic infection with substantial histopathological changes.

### *B*. *pseudohinzii* disrupts auditory functions

**Fig 3:**
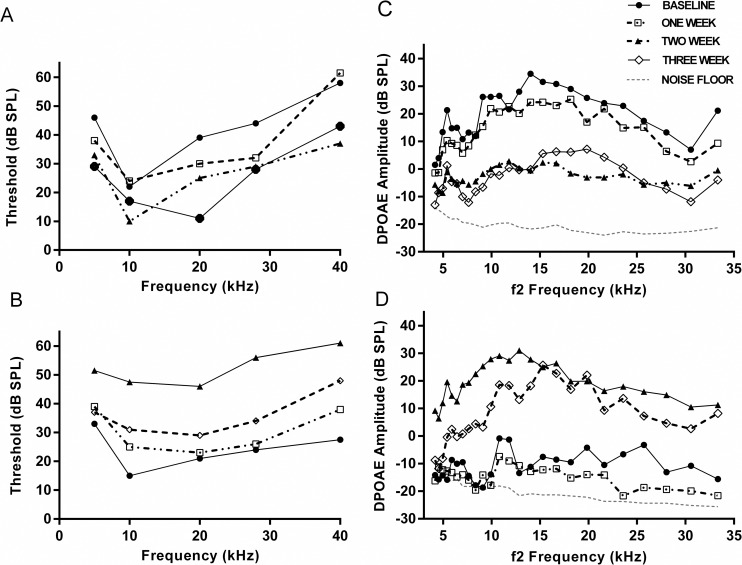
B. pseudohinzii colonization causes measurable hearing loss in C57BL/6J mice. Auditory brainstem response (ABR) thresholds (panel A and B) and distortion product otoacoustic emission (DPOAE) levels (panel C and D) recorded every 3–7 days in mice inoculated with 30 CFU (low dose—panel A and C) or 7500 CFU (high dose- panel B and D) of B. pseudohinzii. Selected time points from individual animals are shown for clarity.

To assess the effects of middle ear infections on auditory function, mice were inoculated with either a low dose (30 CFU) or a high dose (7500 CFU) of *B*. *pseudohinzii* and tested every 3–7 days for hearing loss relative to non-inoculated control animals (Fig 3). The first assay determines the auditory brainstem response (ABR) after tonal stimuli at different frequencies (Fig 3, left panels). An increase in decibels required to elicit a response at any frequency corresponds with a reduction in hearing acuity. No detectible hearing loss was detected 7 dpi for either inoculum. However, two weeks post inoculation with the high dose there were significantly elevated thresholds at all frequencies tested (Fig 3B and 3D) while the low dose resulted in a smaller effect.

A complementary method of measuring hearing loss records differences in Distortion Product Otoacoustic Emissions (DPOAE) (Fig 3, Right panels). Here, the cochlea is stimulated with two simultaneous tone frequencies at a ratio of 1.2, and reductions in the DPOAE correlate with loss of hearing. DPOAE measures of the high dose group revealed significant amplitude depressions one week after inoculation with *B*. *pseudohinzii* for many test frequencies in the 5-15kHz range. Amplitudes were further depressed two weeks post inoculation at all frequencies tested. Similar amplitude depressions were also observed for mice in the low dose group. In summary, using two distinct noninvasive methods to detect hearing loss we observed that *B*. *pseudohinzii* colonization of the middle ear compromises hearing in a dose and time dependent manner, thereby establishing a causal relationship between *B*. *pseudohinzii* colonization and hearing loss.

### *B*. *pseudohinzii* naturally transmits and rapidly reaches the middle ear

**Fig 4:**
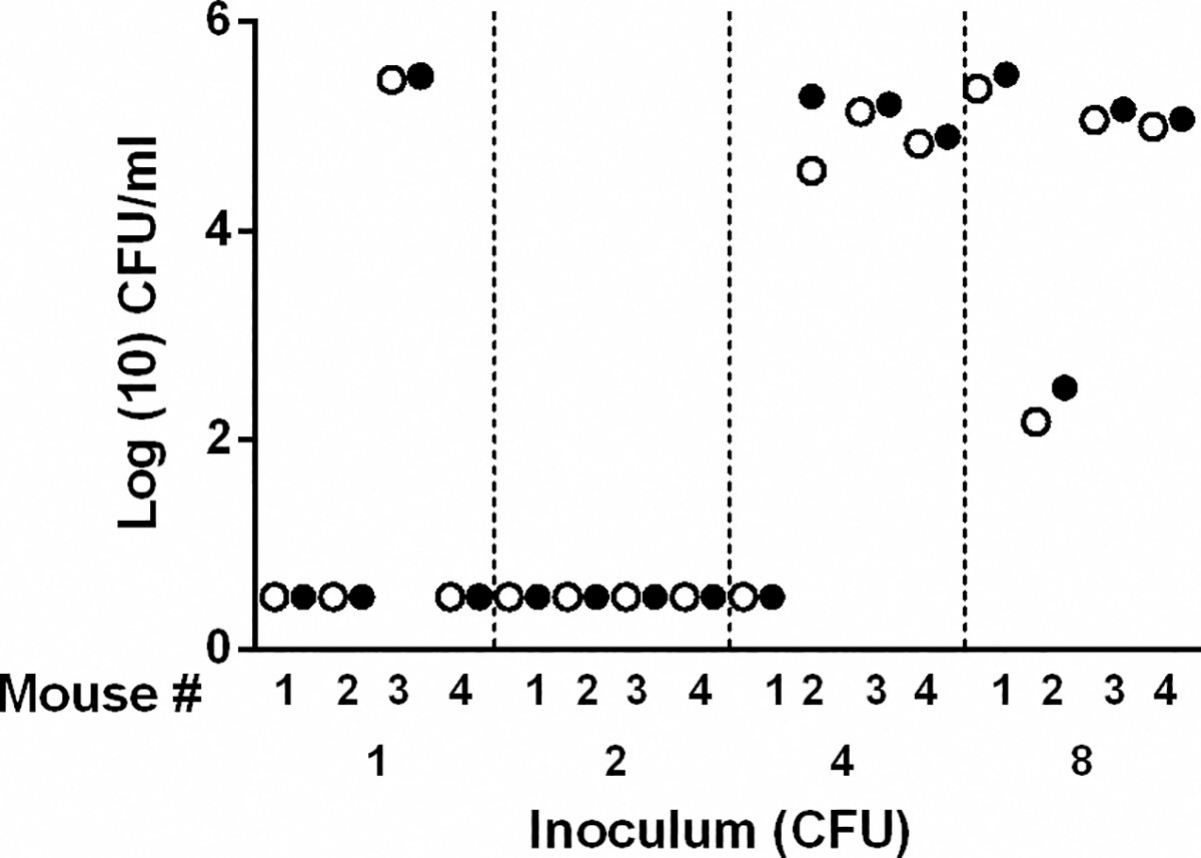
Determining the infectious dose of B. pseudohinzii. The colonization levels of the nasal cavity (filled circles) and middle ears (clear circles) in groups (n = 4) of mice 7 days after being inoculated from the external nares with serial dilutions of an estimated 8, 4, 2 and 1 CFU of B. pseudohinzii in 5 μl PBS.

Being a natural pathogen of the mouse, we reasoned that *B*. *pseudohinzii* might require very few bacteria to establish an infection. To determine the efficiency with which *B*. *pseudohinzii* colonizes mice, we prepared 2-fold dilutions of inocula from an estimated 8 to a limiting 1 CFU and delivered these in a volume of 5 μl of PBS to the external nares of groups of mice. Colonization of the nasal cavity and middle ears of each mouse was assessed after 7 days (Fig 4). Four CFU of *B*. *pseudohinzii* were sufficient to colonize three out of four mice indicating an ID_50_ (Infectious Dose sufficient to colonize 50% of animals) of less than 4 CFU. Interestingly, seven out of the eight mice that became colonized had large numbers (~10^5^) of *B*. *pseudohinzii* in their middle ears and nasal cavity, indicating that the bacterium efficiently reaches and grows within the middle ears.

**Fig 5:**
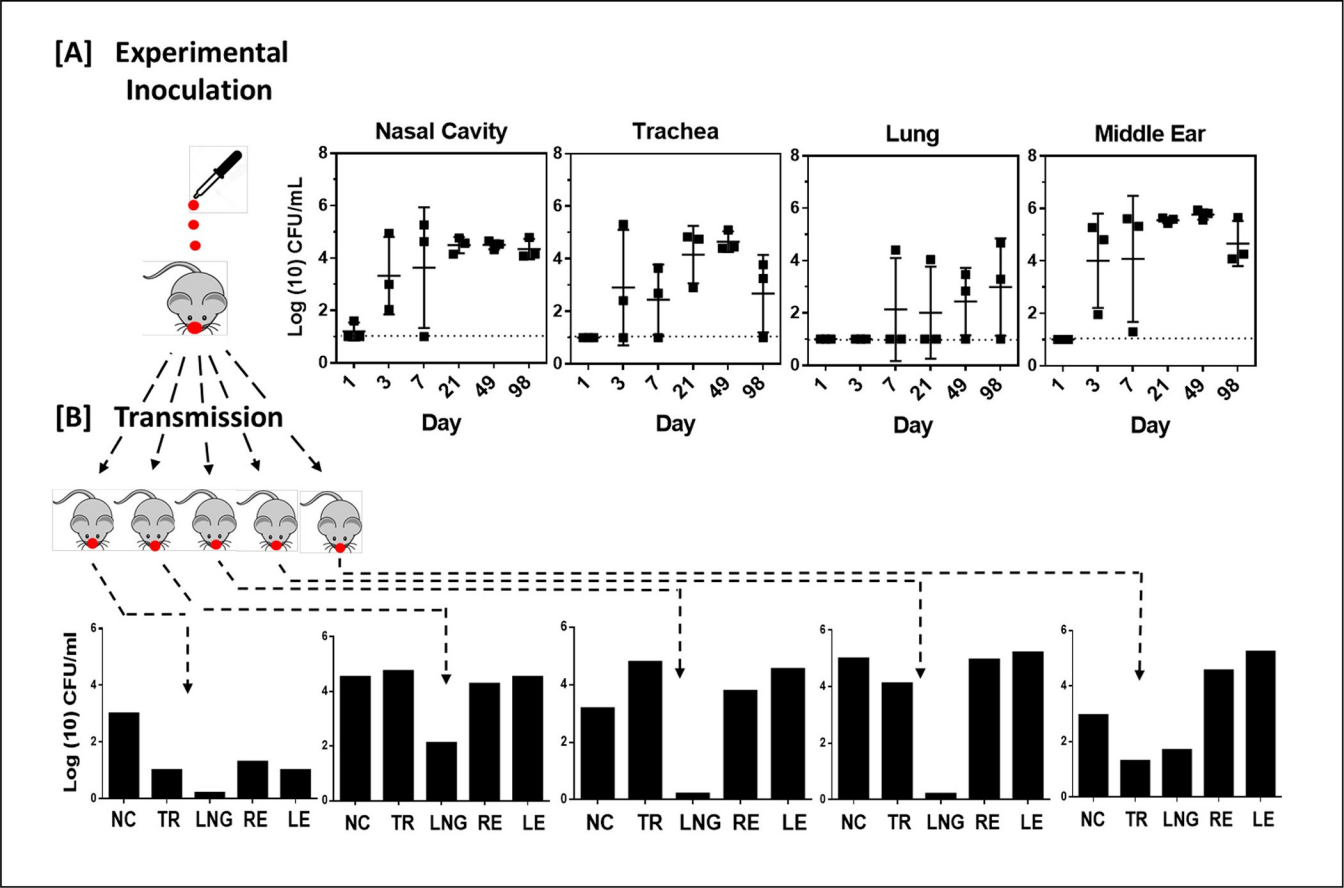
Colonization of mice by B. pseudohinzii following experimental inoculation or natural transmission. A.) Colonization profiles of B. pseudohinzii in the nasal cavity, trachea, lungs and middle ears of C57BL/6J mice experimentally inoculated with B. pseudohinzii (5 CFU in 5 μl) and analyzed on days 1, 3, 7, 21, 49, and 98 days post inoculation (n = 3 per group). Error bars indicate standard deviation. Dashed line indicates the limit of detection. B.) Mice colonized via natural transmission when co-housed for 28 days with experimentally inoculated mice. Colonization profiles for the individual mice are shown in the lower graphs (NC: nasal cavity, TR: trachea, LNG: lungs, RE/LE: right and left middle ear bullae).

To observe the time course of infection in mice inoculated with low dose-low volume of *B*. *pseudohinzii*, simulating natural transmission of small numbers of bacteria, we inoculated groups of 3 C57BL/6J mice with 5 CFU of the bacterium in 5 μl of PBS. We then quantified bacteria in the respiratory tract and middle ears on days 1, 3, 7, 21, 49 and 98 thereafter (Fig 5A). Bacteria were barely detectable on day 1, but then grew steadily to over 10,000 CFU in the nose by day 21 and persisted at about that level thru day 98. A somewhat delayed pattern of spread thru the trachea to the lungs was observed, with more variability in numbers. No bacteria were detected in the middle ears of mice on day 1, but by day 3 every mouse was colonized in the middle ear and by day 21 hundreds of thousands of *B*. *pseudohinzii* were observed in the ears where they stably persisted for over 3 months. These data demonstrate highly efficient and consistent transit up the Eustachian tube to colonize the middle ears of mice between 1 and 3 days post inoculation of the external nares with very low numbers, simulating a natural infection and providing an experimental system in which to carefully study this process.

The spontaneous outbreak of *B*. *pseudohinzii* among wild type C57BL/6J mice noted at separate locations in our mouse facilities indicated that the bacterium was transmissible. To assess the frequency with which *B*. *pseudohinzii* transmission occurred, we used a strategy of co-housing infected (index) and uninfected (naïve) mice to monitor transmission among them. Briefly, we inoculated 2 wild type mice (C57BL/6J) with 75 CFU of *B*. *pseudohinzii* in 5 μl of PBS and introduced 3 naïve mice to the same cage to allow the inoculated index mice to transmit to naïve mice. Transmission was assessed in seven independent cages of co-housed index and naïve mice. We confirmed that the inoculated mice were transmission proficient by monitoring bacterial shedding via swabbing the external nares of the infected mice and enumerating the sampled bacteria on blood agar plates (S2 Fig). After 28 days of co-housing, naïve mice from all 7 cages were sacrificed and their nasal cavities, trachea, lungs and middle ear bullae isolated to monitor bacterial colonization. Within the span of the 28 days, transmission events occurred within 3 of the 7 cages (2 mice each in 2 cages and 1 mouse from another cage) with a total of 5 out of 21 naïve mice (~25%) being colonized. A range of bacterial numbers were recovered from the colonized mice, likely a result of the various times when transmission occurred (Fig 5B). All the mice that had their respiratory tracts colonized also had their middle ears colonized with both left and right bullae of the ears harboring similar numbers of bacteria.

Of all sampled organs, the lungs had the smallest number of bacteria indicating that *B*. *pseudohinzii* does not spread to, colonize and grow in the lower respiratory tract as efficiently as it does the ears. These results demonstrate that *B*. *pseudohinzii* is transmissible under these conditions and rapidly and consistently reaches the middle ears as a part of its infection process. They further indicate that the *B*. *pseudohinzii*-mouse system provides a novel opportunity to study the process of transmission and how that naturally is followed by rapid and consistent ascension of both Eustachian tubes, colonization and growth in both middle ears, where it induces severe inflammatory damage and hearing loss and where it persists for months.

### The *B*. *pseudohinzii* mouse model of otitis media

**Fig 6:**
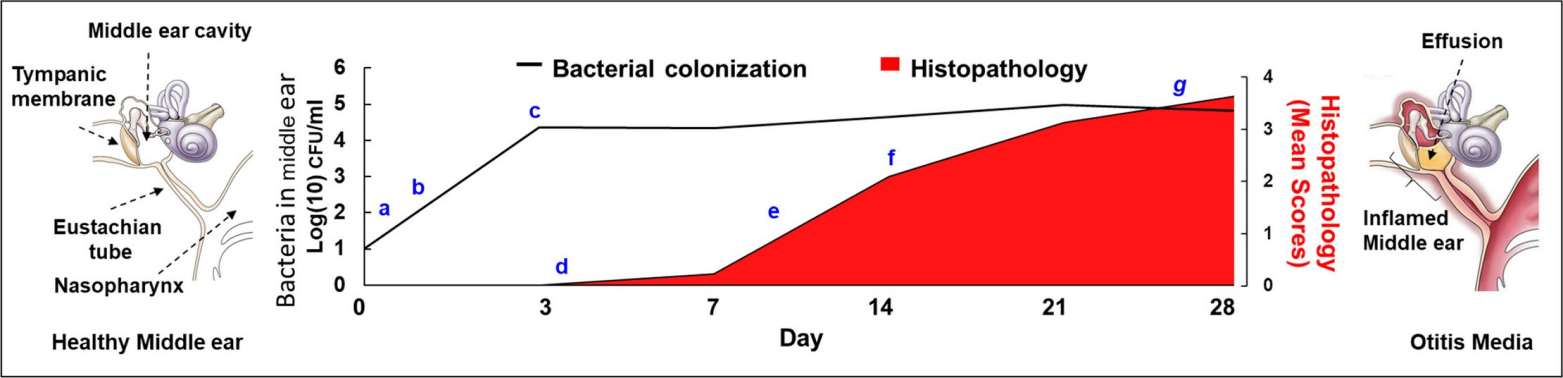
The study of B. pseudohinzii pathogenesis in the middle ear. Figure schematically depicts the timeline in the pathogenesis of B. pseudohinzii induced otitis media in C57BL/6J mice. The bacterial numbers are derived from bacteria recovered from the middle ears, and pathology severity scores [44] are based on histological observations of experimentally infected animals [Histopathologic severity grades—0 (no significant histopathological alterations); 1 (minimal); 2 (mild); 3 (moderate); or 4 (severe)]. Letters (a-g) indicate approximate points where the critical stages in the development of chronic otitis media begin, and which are amenable to study using the B. pseudohinzii-mouse infection model. [a: Nasopharyngeal colonization, b: Ascent of the Eustachian tube, c: Colonization of middle ear, d: Induction of acute inflammatory stage, e: Inflammation and mucoidal effusion, f: Acute inflammatory stage, g: Chronic infection stage].

The *B*. *pseudohinzii*-mouse infection process provides the opportunity to address many basic and important questions about AOM and COM. Fig 6 provides a schematic chronological outline of *B*. *pseudohinzii* infection of the middle ears of mice and the ensuing histopathology, with specific measurable stages indicated by letters a through g. Following natural transmission or experimental inoculation with very low numbers, *B*. *pseudohinzii* can be observed to have rapidly spread to and colonized the middle ear (a). There it grows rapidly in numbers (b) until it reaches over 10^4^ CFU (c). Within days AOM symptoms can be observed histologically via various markers of inflammation (d). These increase progressively, with a measurable pattern and progression of inflammatory stages (e) that contribute to the severe COM and hearing loss (f). Sustained high bacterial numbers likely result in establishment of homeostasis with substantial local pathology, inflammation and hearing loss, consistent with COM (f). Finally, infection ultimately results in immune mediated resolution of infection or persistent, potentially progressive COM (g). Significantly, the indicated stages appear to occur naturally and efficiently following transmission or inoculation, allowing various aspects of each of these steps to be studied and measured using all the tools of mouse molecular immunology to probe the contributions and limitations of each immune function. This experimental system also allows for the detailed measurement of the effects of various interventions (e.g. antibiotics) on the control of chronic middle ear infection.

### Use of deficient mice to define roles of immune cells

**Fig 7:**
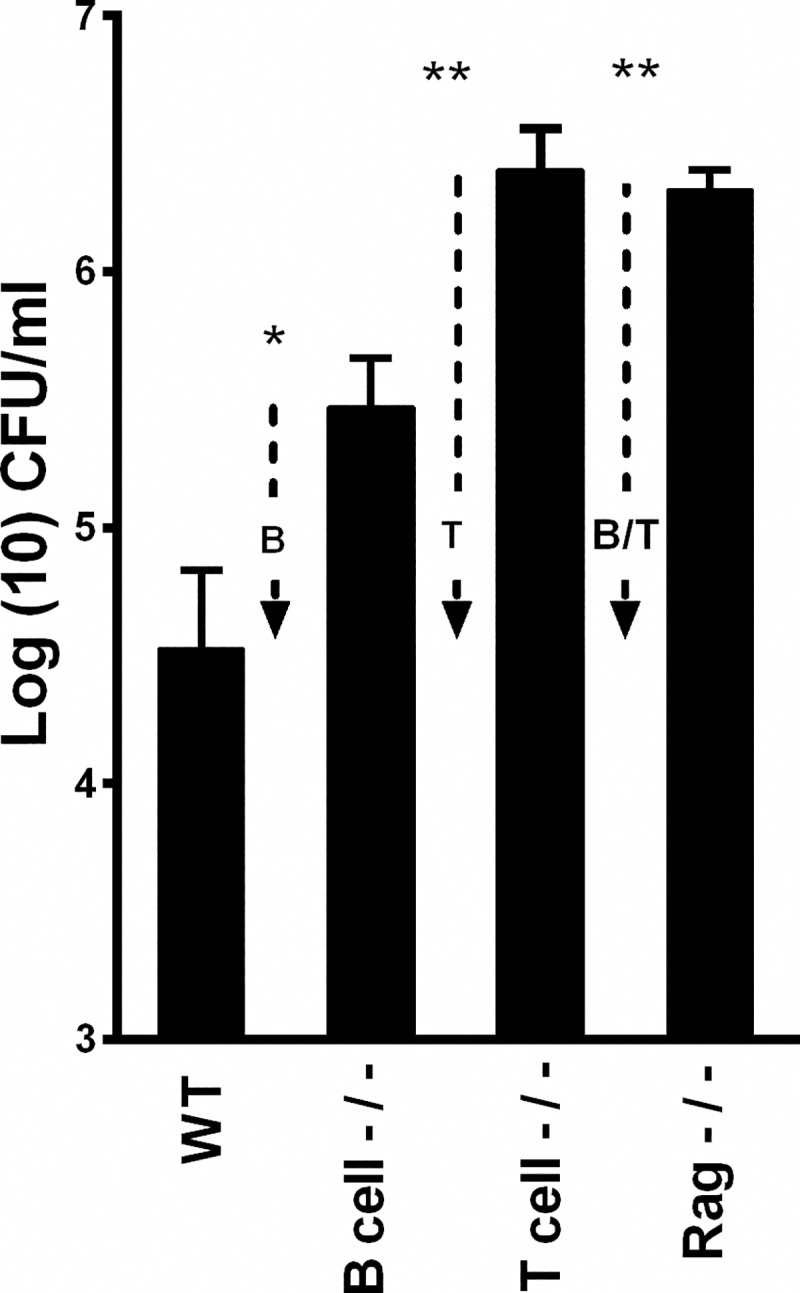
Adaptive immune components contribute to controlling B. pseudohinzii colonization in the middle ears of mice. Numbers of B. pseudohinzii recovered from wild type (C57BL/6J) and B-cell (MuMt-), T-cell (Tcratm1Mom), and B/T-cell (Rag-/-) deficient mice 49 days following inoculation with 7500 CFU delivered to the external nares in 25 μl of PBS. Error bars indicate standard deviation. Arrows indicate the contributions the adaptive immune components have on controlling colonization levels. One-way Anova with Dunnet’s post-test was used to determine statistical difference compared to wild-type: † = p-value <0.1, * = p-value <0.05, ** = p-value<0.002.

The progression of AOM to COM spans the timeframe that can allow the generation of adaptive immunity, but the specific roles of B and T cells are mostly unknown or surmised via correlation. As proof of principle that this experimental system can be used to define the roles of immune cells, we used the well-characterized mouse strains lacking these two cell types. We inoculated *B*. *pseudohinzii* into groups of wild type (WT), B cell deficient (B-), T cell deficient (T-) and B and T cell deficient (B/T-) mice. 49 days later mice were sacrificed, and colonization levels of the middle ears assessed among the separate groups. *B*. *pseudohinzii* colonized the middle ears of wild type mice in moderate numbers (~50,000 CFU), suggesting immune-mediated control of infection (Fig 7). B-cell deficient mice had approximately 10-fold higher numbers of bacteria in the middle ear, revealing an important role for B cells, probably mediated via secreted antibodies. T-cell deficient mice had much higher bacterial numbers, approximately 100-fold than that of WT mice, indicating an even more critical role for T cells in control of bacterial numbers.

Interestingly, the T and B cell deficient mice had numbers that were no higher than T cell deficient mice indicating that in the absence of T cells, B cells have no observable impact on bacterial numbers, consistent with their function being T cell-dependent. These experiments demonstrate that in this experimental system, the tools of mouse molecular immunology can be used to dissect how the immune system works to combat, control, clear, or possibly to contribute to the pathology of OM.

## Discussion

Our limited understanding of how pathogens induce AOM in humans is largely based on current experimental approaches that directly inject large doses of human pathogens into the middle ears of rodents inducing a short-lived acute inflammatory response. Any bacterial pathogen delivered in that way is likely to generate some pathology but the relevance to aspects of human disease may be debated. Further, direct inoculation of the middle ear omits critical steps of a natural infection, including early nasopharyngeal colonization and ascension of the Eustachian tube, adherence/colonization of the middle ear, evasion of the innate immune-defenses and subsequent evasion of the adaptive immune response to establish a chronic infection. So, such systems, although they may use important human pathogens, cannot provide insight to any of these critical aspects of the infectious process.

Here we have shown that *B*. *pseudohinzii* naturally transmits among mice, induces acute inflammation of the middle ears accompanied by decreases in hearing acuity and histopathological changes typically observed in human AOM. Furthermore, the bacterium persists in the middle ear chronically, showing evidence of remodeling of bone tissue and the genesis of lymphoid follicles, features that are currently difficult to induce using infection models of OM. *B*. *pseudohinzii* infection of mice therefore presents itself as a novel model with which many questions associated with the bacterial pathogenesis in OM can be addressed.

While findings in this experimental system may not be directly applicable to specific human pathogens, it presents a unique opportunity to dissect the mechanisms of both bacterial virulence and host immunology in the context of natural infectious OM.

A critical initiating step towards AOM requires that pathogens colonizing the nasopharynx reach the middle ear by ascending the Eustachian tube. Low dose inocula (<10 CFU) of *B*. *pseudohinzii* administered to mice in a 5 μl droplet that deposits bacteria only in the nose are sufficient to colonize the nasal cavity and rapidly and consistently ascend to colonize the middle ears within 3 days, providing an experimental system to examine how pathogens in the nasopharyngeal cavity traverse the Eustachian tube to reach and colonize the middle ear despite the considerable physical and innate immune barriers. As depicted in Fig 6, a number of distinctive stages beyond initial colonization can be monitored and measured in this system, allowing the contribution of individual bacterial factors in each stage to be carefully quantified. The middle ear is an inhospitable environment for microbes, being lined with mucus containing a variety of antimicrobials such as lysozyme, defensins, SPLUNC1 and chitinase [35]. It remains under constant surveillance by Pathogen Recognition Receptors (PRRs) including Toll like receptors, NOD-like receptors and C-type lectins that are poised to recognize a variety of Pathogen Associated Molecular Patterns. The detection of pathogens initiates the cascade of signaling pathways (MAPK/ NF-kB) that culminates in the recruitment of effectors including neutrophils, macrophages, dendritic cells, mast cells, and natural killer cells. The ability to avoid clearance by all of these is common to those bacteria that cause COM and the fact that *B*. *pseudohinzii* successfully persists indicates that it also shares these abilities. There is substantial information available on the virulence mechanisms and comparative strategies used by the member species of the Bordetellae during host immune evasion, and these will greatly facilitate our understanding of *B*. *pseudohinzii* immune evasion strategies and how they operate in the environment of the middle ear.

Applying the methods of mouse immunology allowed us to demonstrate the different roles of specific immune cells in controlling persistent infection of the middle ear. The observation that B cell and T cell deficient mice had significantly larger numbers of bacteria in the middle ear indicates that both B and T cell responses, are important in the control of bacterial numbers, but are not sufficient to clear the infection. These observations are in agreement with earlier descriptions of patients with humoral antibody defects that are particularly prone to middle ear infections [36, 37]. Mouse molecular tools can now define how each of many host immune functions might be involved in discreet stages of OM, from the pattern recognition receptors that might sense initial infection (Fig 6A), through the regulatory cells and molecules involved in the inflammatory response and the ultimate maturation of an adaptive response (Fig 6B).

The clonal spread of pathogens associated with AOM has been documented among siblings suffering from AOM [38, 39]. However, the lack of suitable models has meant that few studies have been conducted on transmission of infectious OM. In our initial observations of the *B*. *pseudohinzii*-mouse experimental system we observed substantial transmission amongst cohoused wild type mice. The moderate rate observed allows room for analysis of interventions that either increase or decrease transmission. A list of conditions could be tested for their ability to contribute to more rapid transmission, such as poor hygiene, old/young age, co-infection or induction of respiratory symptoms. Similarly, this experimental system could be used to test interventions that might decrease or block transmission such as limiting types of contact, antibiotic treatment, vaccination, humidity or resident microbiota of the respiratory tract and ear [40, 41, 42]. Manipulation of specific bacterial factors will also allow the study of the critical molecular mechanisms involved in promoting or mediating transmission of infectious OM.

The most promising aspect of the *B*. *pseudohinzii*-mouse model is the potential of the combination of bacterial genetic and mouse molecular immunological tools to study OM. The related species *Bordetella bronchiseptica* [43], a broad host range respiratory pathogen, also colonizes the middle ears of mice but then does not persist, providing an opportunity for comparative genomics approaches to correlate specific gene subsets with the ability to evade host immunity to allow long term persistence. Such studies will guide reverse genetic approaches to define molecular mechanisms involved in various aspects of these processes. Simultaneously, immune deficient mice can be used to investigate host factors that contribute to the persistence and pathology of the bacterium. The combination of these tools is likely to reveal a great deal about the function of various aspects of the immune system within the ear, and bacterial mechanisms to evade them.

## Materials and methods

### Ethics statement

This study was carried out in accordance with the recommendations in the Guide for the Care and Use of Laboratory Animals of the National Institutes of Health. The protocol was approved by the Institutional Animal Care and Use Committees at The Pennsylvania State University at University Park, PA (#46284 Bordetella-Host Interactions) and at The University of Georgia at Athens, GA (A2016 02-010-A13 Host-Pathogen Interactions, A2016 04-019-A7 Microbiota and Host-Pathogen Interactions, A2016 07-006-A5 Breeding Protocol), or by the Washington University Animal Studies Committee (20090049). Mice were consistently monitored for signs of distress over the course of the experiments to be removed from the experiment and euthanized using carbon dioxide inhalation to prevent unnecessary suffering.

### Bacterial strains and growth

*B*. *pseudohinzii* (strain 8-296-03) has been previously described [34]. *B*. *pseudohinzii* was grown and maintained on Bordet-Gengou (BG) agar (Difco) supplemented with 10% defibrinated sheep’s blood (Hema Resources). Cultures were stored in 25% glycerol at -80°C. For mouse inoculations, *B*. *pseudohinzii* was grown at 37°C, with shaking, to mid-log phase in Stainer Scholte liquid broth (SS). Numbers of bacterial Colony Forming Units (CFU) were estimated by measuring the optical density at 600 nm of *B*. *pseudohinzii* grown in SS media and validated by dilution in phosphate buffered saline (PBS) and plating on BG agar and counting viable colonies after incubation for 2 days at 37°C.

### Mouse experiments

Four- to six- week old C3H/HeJ (000659), C57BL/6J (000664), C57BL/6J-Bcell-/-(MuMt^-^, 002288), C57BL/6J-Tcell-/-(Tcra^tm1Mom^, 002116), C57BL/6J-Rag-/- (002216), and BALB/cJ (000651) mice were procured from The Jackson Laboratory (Bar Harbour, ME) or were provided by the Harvill lab mouse breeding colony, whose mating pairs were also originally purchased from The Jackson Laboratory (University of Georgia, GA). All mice were maintained in specific pathogen-free facilities at the Pennsylvania State Laboratory (University Park, PA) or at the University of Georgia (Athens, GA). All experiments were conducted following institutional guidelines. Mice were lightly sedated with 5% isoflurane (IsoFlo, Abbott Laboratories) and inoculated (1, 2, 4, 8, 75, 150 CFU in 5 μL PBS, or 7500 CFU in 25 μL PBS as per experiment) by pipetting the inoculum as droplets onto their external nares to be inhaled. To quantify bacterial numbers colonizing the respiratory tract and peripheral auditory system, mice were euthanized via CO_2_ inhalation and the organs were excised. Tissues were homogenized in 1 ml PBS, serially diluted, and plated on BG agar. Colonies were counted (n = 3 or 4 per group) following incubation for two days at 37°C.

### Shedding and transmission

Two C57BL/6 mice to be used as index mice were inoculated with 150 CFU *B*. *pseudohinzii* in 5 μl PBS and co-housed with three naïve mice for 28 days. Transmission was assessed in 7 independent cages with 2 index: 3 naïve mice (n = 7). To assess shedding, the external nares of the Index mice were swabbed (30 swipes) with a Dacron-polyester swab starting at day 3 p.i. and every 2 days thereafter. The swabs were vortexed vigorously in 1 ml PBS and bacteria shed from the nasal cavity enumerated on BG agar. To monitor transmission after 28 days of being co-housed, naive mice were sacrificed and the bacterial loads in the middle ear bullae and respiratory tracts were determined as described above.

### Histology

Forty-eight, 5-week-old, female, C57BL/6J mice were divided into two groups and inoculated intranasally with either 25 μl of PBS or with 25 μl containing 7,500 CFU *Bordetella pseudohinzii*. Mice were assessed (n = 6) histopathologically on days 3, 7, 14, 28 and 123 post infection. Following fixation in neutral-buffered, 10% formalin solution and subsequent decalcification in Kristensen’s solution, coronal sections were made through the nose and brain, and transverse sections were made through the middle and inner ear. Tissues were subsequently processed, embedded in paraffin, sectioned at approximately 5 mm, and stained with hematoxylin and eosin. Histopathological exam consisted of evaluation of the nose, brain, and ear for the incidence (presence or absence), severity, and distribution of inflammation. Histopathologic severity scores were assigned as grades 0 (no significant histopathological alterations); 1 (minimal); 2 (mild); 3 (moderate); or 4 (severe) based on an increasing extent and/or complexity of change following Kenneth *et al* [44], unless otherwise specified. Lesion distribution was recorded as focal, multifocal, or diffuse, with distribution scores of 1, 2, or 3, respectively.

### Auditory brainstem response (ABR) thresholds

Mice were anesthetized via intraperitoneal administration of ketamine (80 mg/kg) and xylazine (15 mg/kg). Functional measurements were completed in succession every 3–7 DPI, allowing each animal 2 days of recovery between testing. Procedures were carried out as previously described by Fernandez et al [45]. Briefly, stimulus presentation and data acquisition were performed with TDT System 3 equipment using SigGen and BioSig software (Tucker Davis Technologies, Alachua FL 32615). An ES-1 electrostatic speaker was placed 7 cm from the animal’s right ear. Tone burst stimuli (5, 10, 20, 28 and 40 kHz) were 5 ms in length with a 1 ms rise/fall time and presented at decreasing intensities in 5 dB steps until Wave I was no longer observed. Auditory profiles were recorded using platinum subdermal needle electrodes (Grass Technologies, West Warwick, RI) placed with the recording electrode behind the right pinna, reference electrode at the vertex, and ground electrode in the skin of the back. Responses were amplified (X 100,000) and filtered (100–3,000 Hz) using a Grass P55 pre-amplifier and averaged for 1000 tone bursts at each frequency. Stimulus levels were calibrated using the SigCal program with an ACO Pacific ¼ inch microphone.

### Distortion product otoacoustic emissions (DPOAE)

Signal generation and response recording were controlled with the “EMAV” program (“emission averager”) [46]. Calibration signals (chirps) and primaries (pure tones) were generated using a Lynx L-22 PCI card (Lynx Studio Technology, Inc.). Signals were generated with a 192-kHz sampling rate and routed to an electrostatic speaker driver (ED-1; TDT). Each primary was presented through a separate electrostatic coupled speaker. The speakers were mounted on a customized board alongside an assembly containing a probe microphone (Knowles Electronic series FG-23652-C36), which was inserted into the ear canal of a mouse. The output of the probe microphone was routed to an amplifier (MA-3; TDT) and then to the input of the Lynx L-22 board, where it was sampled at a 192-kHz rate. Dp-grams were collected using two stimuli, at frequencies of f1 and f2, presented with a frequency ratio of f2/f1 = 1.2. Stimulus levels (L1 and L2) were 75 dB SPL and 65 dB SPL, respectively. Each primary pair was presented 48 times for averaging of the DPOAE and noise levels. A frequency step of 1/8 octave was used over a range of f2 frequencies from 5 kHz to 40 kHz. EMAV data files were analyzed using Microsoft Excel.

### Statistics

For experiments determining differences in bacterial loads in the organs of mice the following statistical analysis were performed using GraphPad PRISM (GraphPad Software, Inc): Two-tailed unpaired Student t-tests, One-Way Anova with multiple comparisons and Tukey post-test, and One-Way Anova with Dunnet’s post-test to determine statistical differences compared to wild-type. The specific test used is indicated in the Figure Legends. ABR and DPOAE measures from infected animals were analyzed using a three-way analysis of variance (ANOVA) to compare baseline values and values at 7 and 14 dpi for both low and high dose groups. Two-way ANOVA was subsequently run for each dosage for a closer examination of post-inoculation interval and interactions between this interval and stimulus frequency. Since a large number of frequencies were examined in the DPOAE testing, only every fifth frequency was selected for statistical analysis. A Holm-Sidak method was used for all multiple comparisons. All tests were run in SigmaStat (Systat Software Inc., San Jose, CA).

## Supporting information

S1 Fig *B. pseudohinzii* colonizes the respiratory tract and middle ears of BALB/cJ and C3H/HeJ mice. Graph plots the number of *B*. *pseudohinzii* recovered from the nasal cavity, trachea, lungs and middle ears of BALB/cJ and C3H/HeJ mice 7 DPI after administration of 7500 CFU of *B*. *pseudohinzii* onto the external nares Statistical significance determined by Two-tailed unpaired Student t-test: * = p-value of 0.01, ** = p-value of 0.008, *** = p-value of 0.0007. Error bars indicate standard deviation. Dotted lines indicate limit of detection.
(DOCX)

S2 Fig Shedding profile of *B. pseudohinzii* from C57BL/6 mice. Graphs depict the number of *B*. *pseudohinzii* CFU recovered from individual nasal swabs of inoculated index mice (n = 14) from 3 to 40 days post inoculation. Index mice had been inoculated with ~75 CFU of *B*. *pseudohinzii* in 5 ul PBS. Two infected index mice in each cage (total 7 cages) were co-housed with 3 naïve mice for 28 days. All inoculated index mice shed *B*. *pseudohinzii* (starting after day 3 post inoculation) indicating that they had been colonized and were transmission proficient. Following 28 days of being co-housed transmission of *B*. *pseudohinzii* from index to naïve mice was observed in cages 1 (2 mice), 5 (1 mouse) and 6 (2 mice).
(DOCX)

S1 Table Comparative histopathology scores for the Nasal cavities and Middle ears of C57BL/6J mice that were either uninfected or infected with *B. pseudohinzii* (n = 6). Severity scoring criteria used are derived from Kenneth *et al* (2018) “Use of severity grades to characterize histopathological changes. Toxicol Pathol 46; 256–265”. pmid: 29529947
(DOCX)

S1 Data Data from 70-day colonization profile of *B. pseudohinzii* (Fig 1). (XLSX)

S2 Data Data for ABR Thresholds of mice infected with *B. pseudohinzii* (Fig 3A and 3B). (XLSX)

S3 Data Data for DPOAE of *B. pseudohinzii* infected mice (Fig 3C and 3D). (XLSX)

S4 Data Data for Determination of ID50 of *B. pseudohinzii* in mice (Fig 4). (XLSX)

S5 Data Data for 98-day mouse ear colonization of *B. pseudohinzii* (Fig 5A). (XLSX)

S6 Data Data for ear colonization of mice transmission (Fig 5B). (XLSX)

S7 Data Data for ear colonization of wild type and immunodeficient mice (Fig 7). (XLSX)

S8 Data Data for *B. pseudohinzii* colonization of BALB/CJ and C3H/Hej mice (S1 Fig). (XLSX)
