# Correction: A model of chronic, transmissible Otitis Media in mice

**DOI:** 10.1371/journal.ppat.1007916

**Published:** 2019-06-28

**Authors:** Kalyan K. Dewan, Dawn L. Taylor-Mulneix, Laura L. Campos, Amanda L. Skarlupka, Shannon M. Wagner, Valerie E. Ryman, Monica C. Gestal, Longhuan Ma, Uriel Blas-Machado, Brian T. Faddis, Eric T. Harvill

The eighth author’s name is spelled incorrectly. The correct spelling is: Longhuan Ma.
