# Supplementary material for: A model of chronic, transmissible Otitis Media in mice
Source: PLoS Pathog. 2019 Apr 10;15(4):e1007696. doi: 10.1371/journal.ppat.1007696 (PMC6476515; doi:10.1371/journal.ppat.1007696)
Supplement: S1 Fig — Graph plots the number of B. pseudohinzii recovered from the nasal cavity, trachea, lungs and middle ears of BALB/cJ and C3H/HeJ mice 7 DPI after administration of 7500 CFU of B. pseudohinzii onto the external nares Statistical significance determined by Two-tailed unpaired Student t-test: * = p-value of 0.01, ** = p-value of 0.008, *** = p-value of 0.0007. Error bars indicate standard deviation. Dotted lines indicate limit of detection. (DOCX) [file ppat.1007696.s001.docx]

**S1_Fig.**


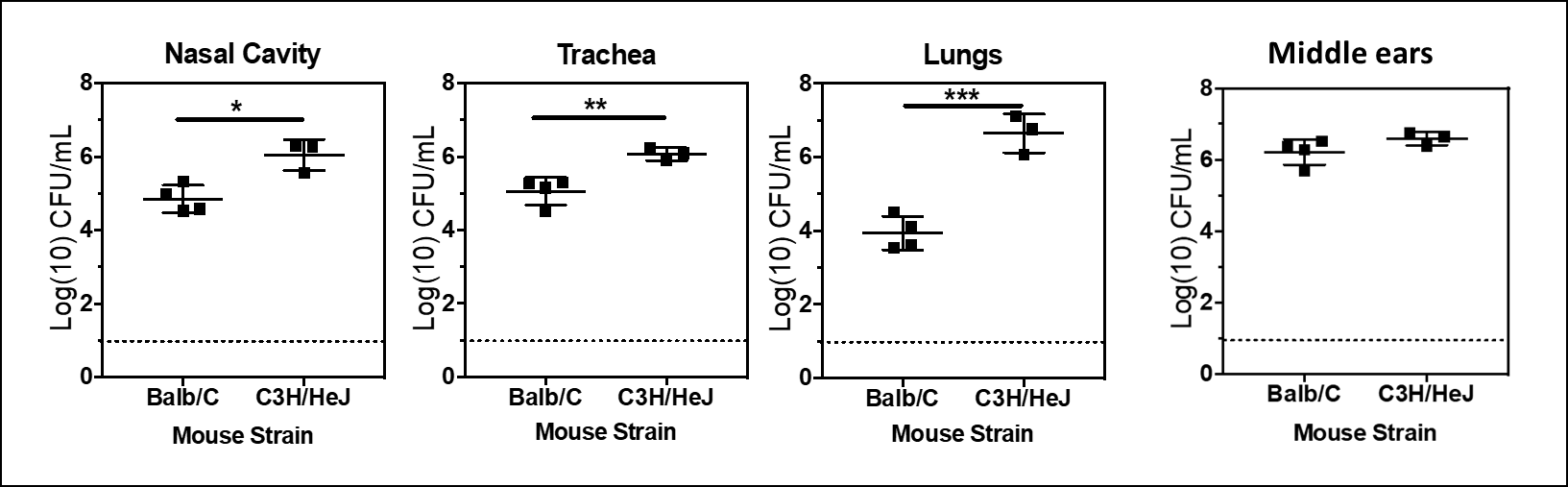


***B. pseudohinzii* colonizes the respiratory tract and middle ears of Balb/C and C3H/HeJ mice.**

Graph plots the number of *B. pseudohinzii* recovered from the nasal cavity, trachea, lungs and middle ears of Balb/C and C3H/HeJ mice 7dpi after administration of 7500 CFU of *B. pseudohinzii* onto the external nares Statistical significance determined by Two-tailed unpaired Student t-test: *=p-value of 0.01, **=p-value of 0.008, ***=p-value of 0.0007. Error bars indicate standard deviation. Dotted lines indicate limit of detection.
