# Supplementary material for: A model of chronic, transmissible Otitis Media in mice
Source: PLoS Pathog. 2019 Apr 10;15(4):e1007696. doi: 10.1371/journal.ppat.1007696 (PMC6476515; doi:10.1371/journal.ppat.1007696)
Supplement: S2 Fig — Graphs depict the number of B. pseudohinzii CFU recovered from individual nasal swabs of inoculated index mice (n = 14) from 3 to 40 days post inoculation. Index mice had been inoculated with ~75 CFU of B. pseudohinzii in 5 ul PBS. Two infected index mice in each cage (total 7 cages) were co-housed with 3 naïve mice for 28 days. All inoculated index mice shed B. pseudohinzii (starting after day 3 post inoculation) indicating that they had been colonized and were transmission proficient. Following 28 days of being co-housed transmission of B. pseudohinzii from index to naïve mice was observed in cages 1 (2 mice), 5 (1 mouse) and 6 (2 mice). (DOCX) [file ppat.1007696.s002.docx]

**S3_Fig**


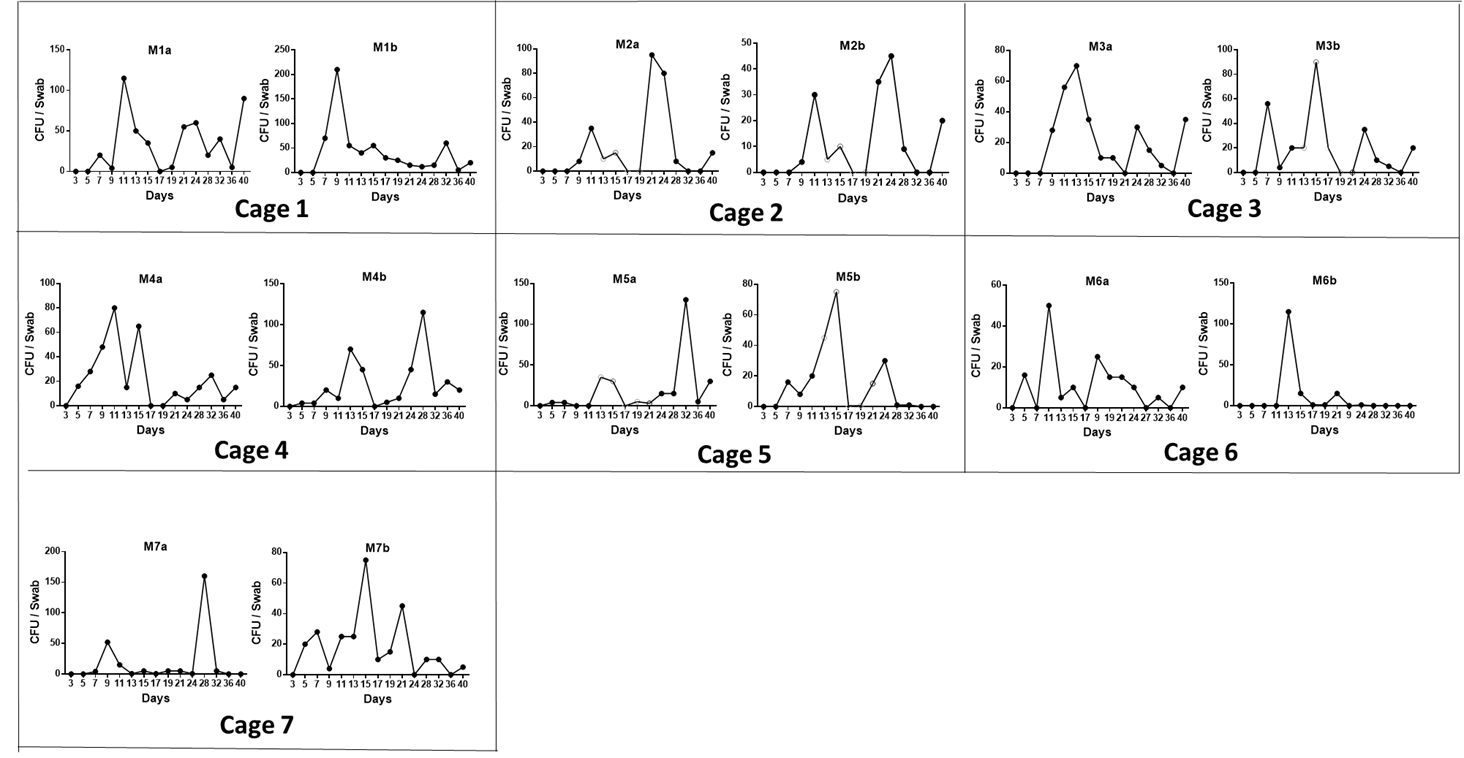


**S2 Fig. Shedding profile of *B. pseudohinzii* from C57Bl/6 mice.**

Graphs depict the number of *B. pseudohinzii* CFU recovered from individual nasal swabs of inoculated index mice (n=14) from 3 to 40 days post inoculation. Index mice had been inoculated with ~75 CFU of *B. pseudohinzii* in 5ul PBS. Two infected index mice in each cage (total 7 cages) were co-housed with 3 naïve mice for 28 days. All inoculated index mice shed *B. pseudohinzii* (starting after day 3 p.i.) indicating that they had been colonized and were transmission proficient. Following 28 days of being co-housed transmission of *B. pseudohinzii* from index to naïve mice was observed in cages 1 (2 mice), 5 (1 mouse) and 6 (2 mice).
