# Supplementary figures and images for: A model of chronic, transmissible Otitis Media in mice
Source: PLoS Pathog. 2019 Apr 10;15(4):e1007696. doi: 10.1371/journal.ppat.1007696 (PMC6476515; doi:10.1371/journal.ppat.1007696)

**
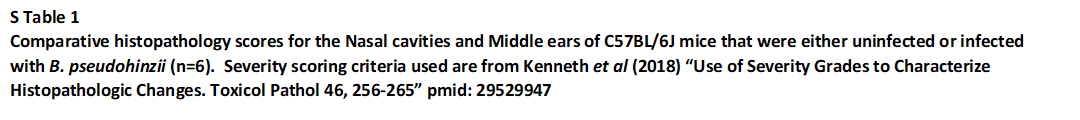
**

Supplement: S1 Table — Severity scoring criteria used are derived from Kenneth et al (2018) “Use of severity grades to characterize histopathological changes. Toxicol Pathol 46; 256–265”. pmid: 29529947 (DOCX) [file ppat.1007696.s003.docx]
